# Detection of microbial cell-free DNA in maternal and umbilical cord plasma in patients with chorioamnionitis using next generation sequencing

**DOI:** 10.1371/journal.pone.0231239

**Published:** 2020-04-15

**Authors:** Russell G. Witt, Lily Blair, Michela Frascoli, Michael J. Rosen, Quoc-Hung Nguyen, Sivan Bercovici, Simona Zompi, Roberto Romero, Tippi C. Mackenzie

**Affiliations:** 1 Eli and Edythe Broad Center of Regeneration Medicine, University of California, San Francisco, California, United States of America; 2 Department of Surgery, University of California, San Francisco, California, United States of America; 3 Karius Inc., Redwood City, California, United States of America; 4 D2G Oncology, Inc: Mountain View, California, United States of America; 5 Department of Experimental Medicine, School of Medicine, University of California, San Francisco, California, United States of America; 6 Wayne State University, Detroit, Michigan, United States of America; 7 Perinatology Research Branch, National Institute of Child Health and Human Development/National Institutes of Health/Department of Health and Human Services, Bethesda, Maryland, and Detroit, Michigan, United States of America; 8 Center for Maternal-Fetal Precision Medicine, University of California, San Francisco, California, United States of America; Shanghai Jiao Tong University, CHINA

## Abstract

**Background:**

Chorioamnionitis has been linked to spontaneous preterm labor and complications such as neonatal sepsis. We hypothesized that microbial cell-free (cf) DNA would be detectable in maternal plasma in patients with chorioamnionitis and could be the basis for a non-invasive method to detect fetal exposure to microorganisms.

**Objective:**

The purpose of this study was to determine whether next generation sequencing could detect microbial cfDNA in maternal plasma in patients with chorioamnionitis.

**Study design:**

Maternal plasma (n = 94) and umbilical cord plasma (n = 120) were collected during delivery at gestational age 28–41 weeks. cfDNA was extracted and sequenced. Umbilical cord plasma samples with evidence of contamination were excluded. The prevalence of microorganisms previously implicated in choriomanionitis, neonatal sepsis and intra-amniotic infections, as described in the literature, were examined to determine if there was enrichment of these microorganisms in this cohort. Specific microbial cfDNA associated with chorioamnionitis was first detected in umbilical cord plasma and confirmed in the matched maternal plasma samples (n = 77 matched pairs) among 14 cases of histologically confirmed chorioamnionitis and one case of clinical chorioamnionitis; 63 paired samples were used as controls. A correlation of rank of a given microorganism across maternal plasma and matched umbilical cord plasma was used to assess whether signals found in umbilical cord plasma were also present in maternal plasma.

**Results:**

Microbial DNA sequences associated with clinical and/or histological chorioamnionitis were enriched in maternal plasma in cases with suspected chorioamnionitis when compared to controls (12/14 microorganisms, p = 0.02). Analysis of the microbial cfDNA in umbilical cord plasma among the 1,251 microorganisms detectable with this assay identified *Streptococcus mitis*, *Ureaplasma* spp., and *Mycoplasma* spp. in cases of suspected chorioamnionitis. This assay also detected cfDNA from *Lactobacillus* spp. in controls. Comparison between maternal plasma and umbilical cord plasma confirmed these signatures were also present in maternal plasma. Unbiased analysis of microorganisms with significantly correlated signal between matched maternal plasma and umbilical cord plasma identified the above listed 3 microorganisms, all of which have previously been implicated in patients with chorioamnionitis (*Mycoplasma hominis* p = 0.0001; *Ureaplasma parvum* p = 0.002; *Streptococcus mitis* p = 0.007). These data show that the pathogen signal relevant for chorioamnionitis can be identified in both maternal and umbilical cord plasma.

**Conclusion:**

This is the first report showing the detection of relevant microbial cell-free cfDNA in maternal plasma and umbilical cord plasma in patients with clinical and/or histological chorioamnionitis. These results may lead to the development of a specific assay to detect perinatal infections for targeted therapy to reduce early neonatal sepsis complications.

## Introduction

Chorioamnionitis is a common complication of pregnancy and affected patients are at risk for maternal and adverse neonatal outcomes (sepsis, endomyometritis, pelvic abscess, early neonatal sepsis, and cerebral palsy) [1–5]. Acute histological chorioamnionitis is defined as an acute inflammation and infiltration of the chorion and amnion, which is often evidenced by neutrophil infiltration [6]. The cause of acute chorioamnionitis was previously thought to be the result of either an ascending bacterial infection, iatrogenically during invasive procedures, or hematogenously [7, 8]. While not always present, certain microorganisms have been associated with the development of acute chorioamnionitis and intra-amnionitic infection (IAI)[9–11][. However, the presence of microorganisms has not consistently been shown and there have been identified subgroups of patients with sterile intra-amniotic inflammation[12–14] and, in clinical chorioamnionitis at term, patients with intra-amniotic inflammation but no detectable microorganism by either culture or bacterial 16S rDNA polymerase chain reaction (37).

In cases of clinical chorioamnionitis, cultures from the mother and offspring are often obtained but commonly do not result pathogen identification nor affect the duration of broad-spectrum antibiotic therapy [15–17]. Subclinical infections may lead to preterm labor or preterm premature rupture of the membranes and do not present with overt symptoms of infection [18]. It remains unclear if the lack of confirmatory culture results is due to limitations in culturing techniques or if sterile inflammatory processes are contributing to preterm labor.

Cell-free DNA (cfDNA) analysis has emerged as a powerful non-invasive diagnostic tool in fields such as oncology, prenatal diagnosis, and infectious diseases [19] [21–23] [24]. cfDNA refers to extracellular circulating nucleic acids, which result from mechanisms such as apoptosis, phagocytosis, or active cell release [20]. Interest in the use of cfDNA during pregnancy began after the discovery of the presence of fetal DNA circulating in maternal plasma [25]. Maternal plasma could then be non-invasively assayed as a potential reflection of fetal physiology or pathology, e.g. in preterm labor, β-thalassemia, and fetal aneuploidy [26–28]. Most recently, we have validated a new laboratory developed clinical diagnostic test, the Karius^®^ Test, and showed that DNA sequencing of cell-free plasma can accurately detect microbial cfDNA in bloodstream infections and deep seated infections [29, 30].

The current study investigates whether microbial cfDNA can be identified in the maternal plasma and the umbilical cord plasma in patients with clinical or histological chorioamnionitis. Using a research use only (RUO) assay developed by Karius Inc., that includes a modification to the validated clinical Karius test to increase sensitivity to microbial signal, we could detect the presence of microbial cfDNA in maternal plasma and umbilical cord plasma in cases of chorioamnionitis.

## Materials and methods

### Study design

Samples for next generation sequencing (NGS) were selected among residual plasma samples with an available volume of at least 50μl, collected from two prospective observational studies at the University of California, San Francisco, CA (UCSF) and Hutzel Women’s Hospital in Detroit, MI, part of the Detroit Medical Center, and a major affiliate of the Wayne State University School of Medicine. Samples for NGS were selected among samples banked between January 15^th^, 2010 to December 8^th^, 2016. The UCSF and Wayne State University Institutional Review Boards approved the studies. Inclusion criteria for the observational studies were: any viable pregnancy, maternal age >18 years, ability to sign informed consent, and intent to deliver and receive antenatal care at either the UCSF or Hutzel Women’s Hospital. Exclusion criteria included: nonviable pregnancies or inability to consent. Patients were followed until delivery. Clinical chorioamnionitis was defined by the presence of maternal fever, and either maternal leukocytosis, uterine fundal tenderness, maternal tachycardia (>100/min), fetal tachycardia (>160/min) and purulent or foul amniotic fluid; and histological chorioamnionitis was defined by neutrophilic infiltration of the chorion.

### Sample collection and processing

Whole blood was collected from patients prior to or shortly after delivery in an EDTA-coated Vacutainer^®^ (Becton Dickson, Franklin Lakes, NJ), processed to plasma by centrifugation (1500 rpm for 10 minutes at room temperature), within 72 hours of collection and stored at -80°C until shipment to Karius, Inc. (Redwood City, CA) for processing and analysis.

### Direct NGS of cfDNA from patient plasma

cfDNA was extracted from plasma, DNA libraries constructed and sequenced on an Illumina NextSeq^®^ 500. Negative controls and positive controls were processed alongside patient samples in every batch. Sequencing reads identified as human were removed, and the remaining sequences were aligned to a curated pathogen database. Abundances for over 1,000 organisms in the Karius clinical reportable range were computed. For detailed methods, see S1 Appendix.

### Graphical display of cluster analysis

Two filters were applied to the data before visualization as a heatmap. First, we removed any samples that were dominated by vaginal or gut commensal microorganisms that likely entered the umbilical cord blood sample during collection. Specifically, we removed samples for which 5 or more microorganisms known to be present in normal gut and/or vaginal flora were seen significantly (with p< = 1e-35) above baseline levels determined by negative samples. The threshold of 5 was identified by comparing the distribution of significance levels for all microorganisms in each sample.

To account for the background level of each microorganism, originating from either the patients or potential environmental contamination, we converted the estimated concentration of each microorganism in each sample into a z-score where the population is considered to be the set of concentrations of that microorganism across all samples. The heatmap presents microorganism concentrations with z-scores greater than 2, i.e. those that are 2 standard deviations above the cohort mean.

In the heatmap, the microorganisms are ordered as consecutive leaves of a tree built from standard taxonomy and the samples were ordered, within preterm and at term births, using Unweighted Pair Group Method with Arithmetic Mean hierarchical clustering using the Pearson’s distance. A black box denotes the microorganism concentrations for which the null hypothesis of having originated from environmental contamination is rejected at a significance threshold of 1e-35.

### Calculating Spearman correlations and p-value plots

We expect microorganisms that are the source of infection to be elevated in the maternal plasma as well as the umbilical cord plasma, whereas the abundance of contaminants should be independent from one sample to another. Therefore, to identify microorganisms causing infection in this set of patients, we calculated for each microorganism the correlation of the abundances in the maternal plasma samples with their paired umbilical cord plasma samples. The measure used for this correlation was a p-value representing the significance of the enrichment of the microorganism above the expected background. The assumption was that the microorganisms driving the infection would be observed with abundances that are significantly above background in both matching samples. To account for the fact that the signal may be weaker in the maternal plasma than the umbilical cord plasma, we used a Spearman rank correlation for this analysis. P-values for the Spearman correlation were ranked and displayed in comparison to a uniform null distribution. A Benjamini-Hochberg correction was applied and thresholds for a false discovery rate of 0.05 and 0.1 were displayed on the plot.

Microorganisms for which the abundance in negative control samples changed significantly due to a reagent lot change were removed from the Spearman correlation analysis. If the mean abundance changed by a factor of at least 2 and a difference of at least 10 molecules per microliter after a lot change, the microorganism was removed from the analysis.

## Results

### Population and clinical characteristics

**Fig 1:**
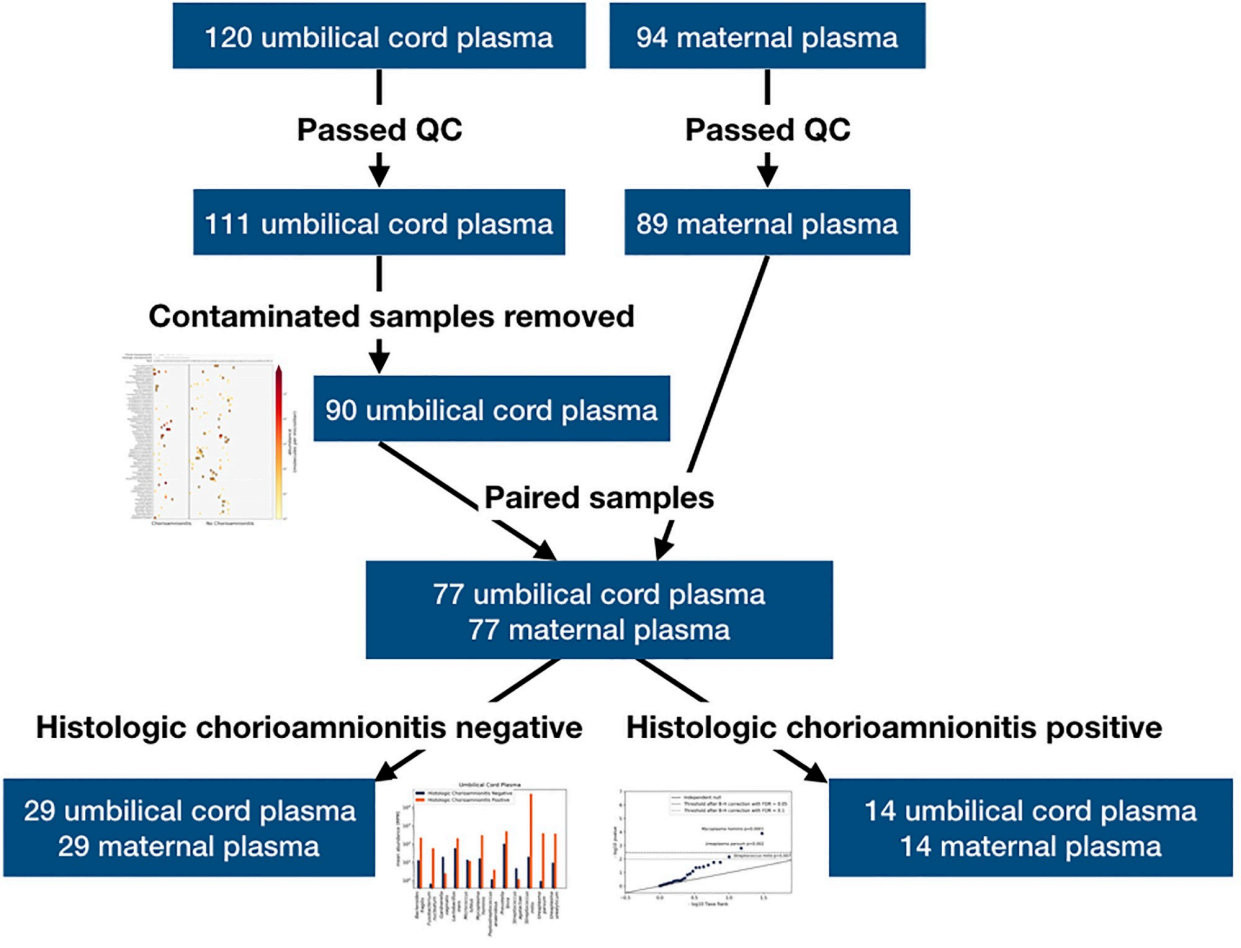
Diagram of sample processing and analysis.

120 umbilical cord plasma and 94 maternal plasma samples were sequenced, among which 111 and 89 passed quality control, respectively (Fig 1). We subsequently discarded 21 umbilical cord plasma samples that appeared to have been contaminated at the draw site (Fig 1), the criterion for which was the presence of 5 or more microorganisms known to be present in normal gut and/or vaginal flora seen significantly above baseline levels as determined by negative control samples. All the 90 remaining umbilical cord plasma samples were used to generate a heatmap, a graphical visualization of cluster analysis. Among these, 77 had a matching maternal plasma sample that returned a valid result (Fig 1). Demographics data of the paired samples are shown in Table 1.

**Table 1:** Demographics data of paired maternal plasma and umbilical cord plasma samples with valid NGS results (n = 77).

	Control (n = 63)	Histologic Chorioamnionitis (n = 14)	*P*-value
Maternal age (y)	32 (27–36)	25 (20–29)	**0.02**
Race/Ethnicity			**0.02**
African American	9 (14%)	5 (36%)	
Caucasian	25 (40%)	1 (7%)	
Hispanic	7 (11%)	2 (14%)	
Asian	9 (14%)	0 (0%)	
Other/Mixed	3 (5%)	0 (0%)	
Unknown	10 (16%)	6 (43%)	
Primigravid	21 (33%)	5 (36%)	0.86
Gestational Age (weeks)	38 (35.9–39.7)	33.1 (31.6–38.2)	**0.01**
Cesarean delivery	22 (35%)	4 (29%)	0.76
Preterm premature rupture of membranes	17 (27%)	8 (57%)	0.05
Birth weight (g)	2978 (2421–3541)	2225 (1790–3250)	**0.03**

### NGS detects microbial cfDNA signatures that differ between patients with and without chorioamnionitis

**Fig 2:**
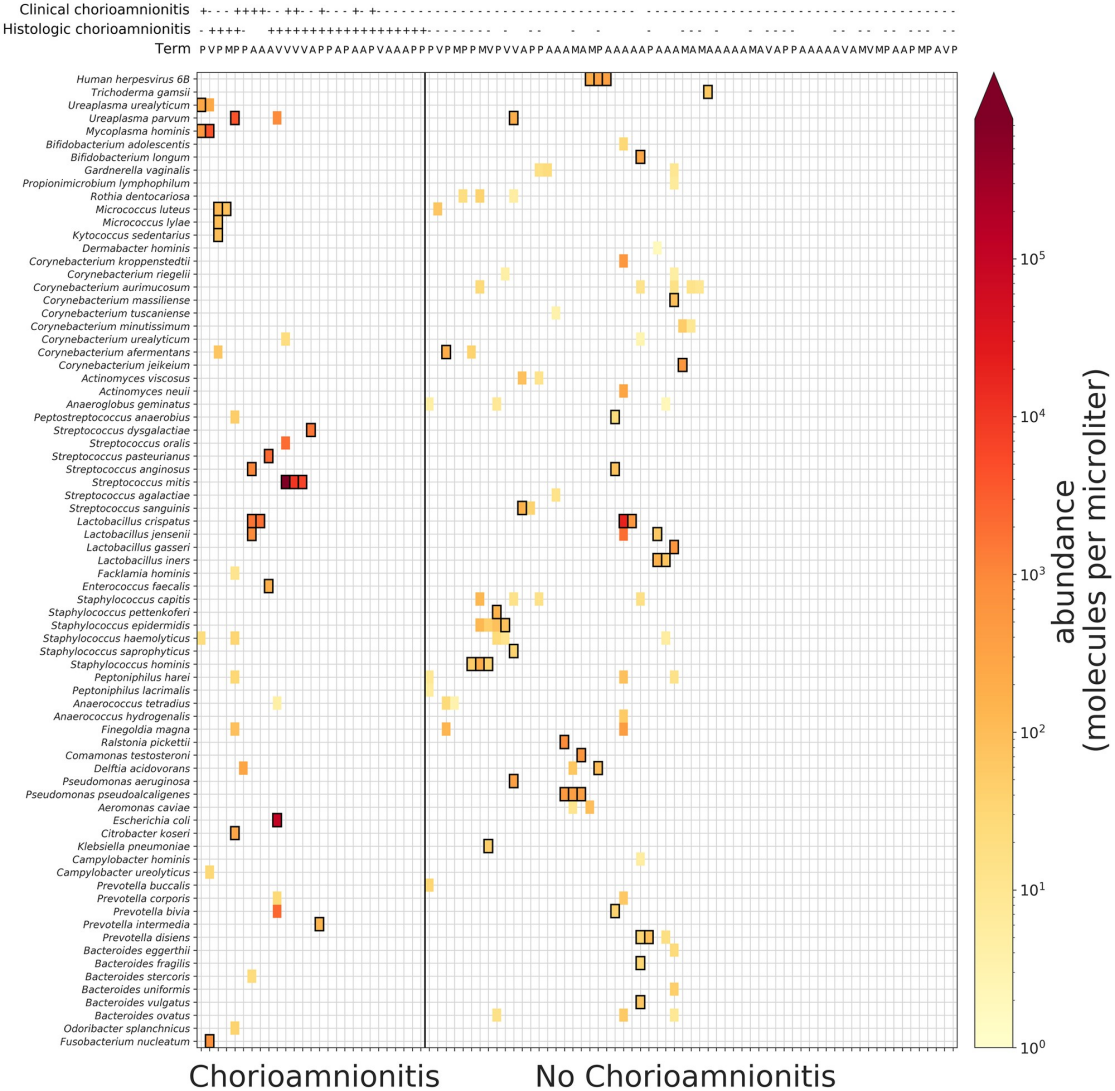
Microbial abundances in umbilical cord plasma detected by next generation sequencing (NGS). Umbilical cord plasma samples that passed quality control and did not appear to be contaminated at the collection site were included in this analysis (n = 90). The microbial abundances of >1,000 organisms detectable with NGS were compared, and microorganisms were clustered and displayed as a heatmap visualization. Only microorganisms that were present at abundances significantly above the negative control baseline for at least one patient were included in the figure, and then any microorganisms in samples for which the abundances were not at least 2 z-scores above the rest of the samples for the same microorganism were filtered out (see Methods). Black boxes represent calls that were significantly above the baseline determined from negative controls. Presence (+) or absence (-) of clinical and histological chorioamnionitis are shown for each subject above the heatmap.

Because the signal of cfDNA is strongest near the site of infection and likely more prevalent within the umbilical cord plasma than maternal plasma, we first assessed the microbial signature of chorioamnionitis in the umbilical cord plasma. The microbial abundances of organisms detectable with this RUO assay were compared and microorganisms at levels above baseline of negative control samples are shown in a heatmap (Fig 2). 27/90 patients had histologic or clinical chorioamnionitis and 63/90 had neither; based on inspection of the heatmap, microbial signatures differ among these groups. In cases of confirmed histological chorioamnionitis (n = 23), we found that signals for *Streptococcus mitis*, *Ureaplasma* spp., and *Mycoplasma* spp. were enriched. Two out of three cases with confirmed histological chorioamnionitis and high abundance of *Streptococcus mitis* also had clinical chorioamnionitis and neonatal sepsis. Neonates with *Streptococcus mitis* were born prior to 32 weeks of gestation. In patients without evidence of chorioamnionitis (n = 63), NGS detected a signal for multiple *Lactobacillus* and *Staphylococcus* spp. A list of all the microorganisms identified at levels considered significant by the clinical validated assay can be found in S1 Table.

### NGS detects cfDNA from relevant microorganisms associated with chorioamnionitis in umbilical cord plasma

**Fig 3:**
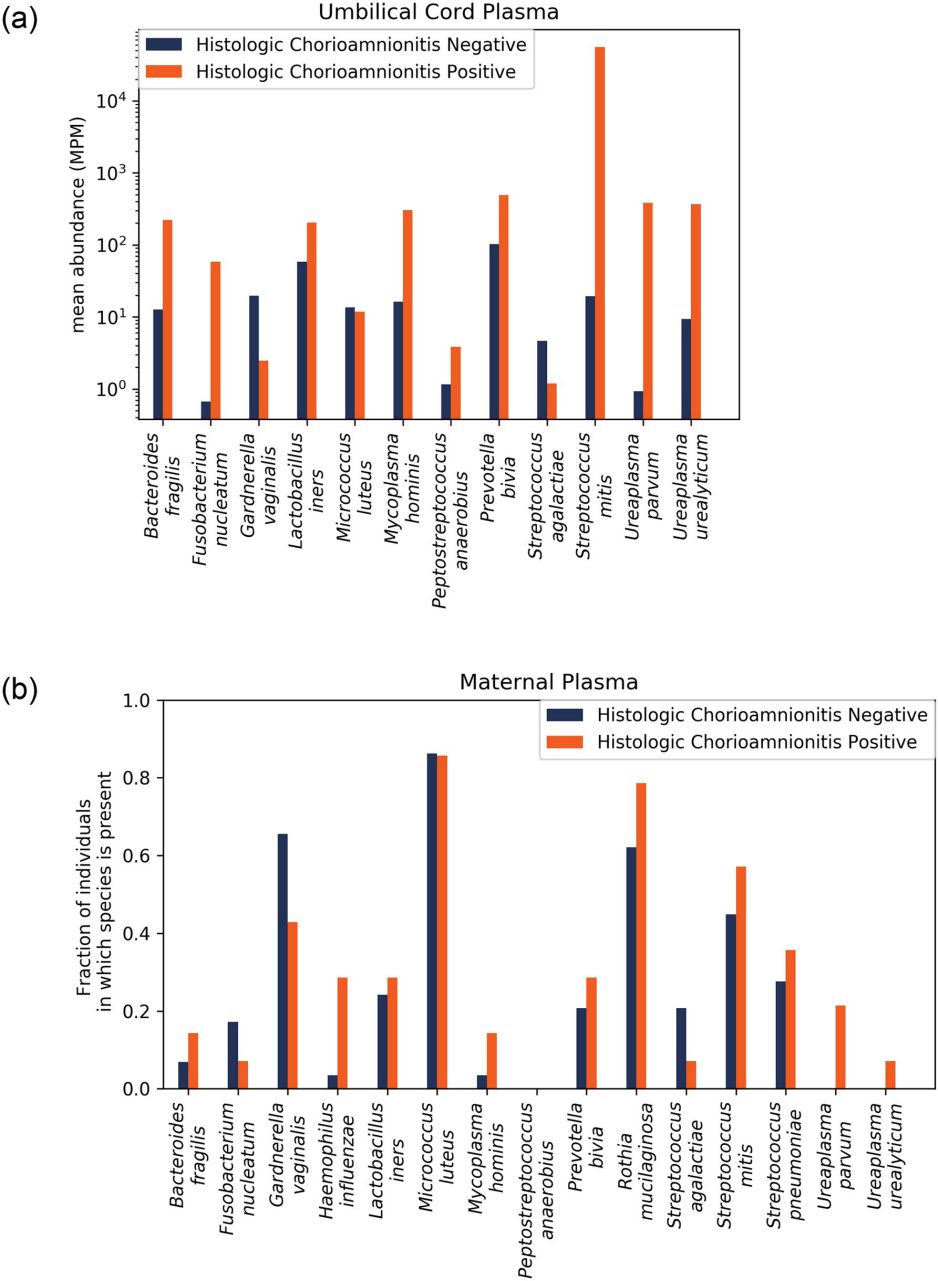
A. Microorganisms associated with chorioamnionitis are enriched in umbilical cord plasma of patients with histological chorioamnionitis. Comparison of the enrichment of microorganisms known to be involved in chorioamnionitis, intra-amniotic infections and preterm birth in cases with histological chorioamnionitis (n = 14) to cases where histology of the placenta was negative for chorioamnionitis (n = 29). B. Microorganisms associated with chorioamnionitis are enriched in maternal plasma of patients with histological chorioamnionitis. The enrichment of this set of microorganisms in maternal plasma is significant (p = 0.02).

We next probed for a set of microorganisms relevant to chorioamnionitis. The microorganisms examined included microorganisms associated in the literature with chorioamnionitis, early onset neonatal sepsis and/or preterm birth [13, 31–33] (Table 2). We calculated the enrichment of each microorganism for all cases that had histological analysis of the placenta available (Fig 1), among which 14 had histological chorioamnionitis and 29 had normal placental histology. In umbilical cord plasma, we calculated the mean abundance for each pathogen across samples in each group. The abundance was enriched in samples with histological chorioamnionitis for 9/12 of the microorganisms, and for *Streptococcus mitis*, the mean abundance was over 3 orders of magnitude higher in the chorioamnionitis group (Fig 3A). A Fisher’s exact test on the binary enrichment for this set of microorganisms was not significant (p = 0.057).

**Table 2:** Microorganisms associated in the literature with chorioamnionitis, early onset neonatal sepsis and/or preterm birth.

*Bacteroides fragilis*
*Fusobacterium nucleatum*
*Gardnerella vaginalis*
*Haemophilus influenzae*
*Lactobacillus iners*
*Microcuccus luteus*
*Mycoplasma hominis*
*Peptostreptococcus anaerobius*
*Prevotella bivia*
*Rothia mucilaginosa*
*Streptococcus agalactiae*
*Streptococcus mitis*
*Ureaplasma parvum*
*Ureaplasma urealyticum*

### NGS detects cfDNA from relevant microorganisms associated with chorioamnionitis in maternal plasma

We then calculated the enrichment based on the number of control maternal samples in which the microorganism was present and represented by at least 1 read (Fig 3B). This differs from the metric used for the umbilical cord plasma, because the expected signal in the maternal plasma is much lower than that of the umbilical cord plasma. We found a significantly increased enrichment for the set of organisms in patients with histological chorioamnionitis (p = 0.02). *Ureaplasma urealyticum*, and *Ureaplasma parvum* were only present in cases of chorioamnionitis.

We hypothesized that the microbial signal is weaker in maternal plasma. In support of this, only 18/89 (20.2%) maternal plasma samples had a positive result, with the presence of microorganisms at a significant level (S1 Table). Among these, 4/89 had a microorganism that was also found in umbilical cord blood plasma and 6/89 maternal samples returned a valid result with one or more microorganisms previously reported in IAI; however, these microorganisms were either not found in the matching umbilical cord plasma or the umbilical cord plasma was not available for analysis. In cases with funisitis present on histology, 3/8 (37.5%) yielded a positive microbial signal (S1 Table).

### Streptococcus mitis, Ureaplasma parvum, and Mycoplasma hominis detected by NGS significantly correlated between umbilical cord plasma and maternal plasma

**Fig 4:**
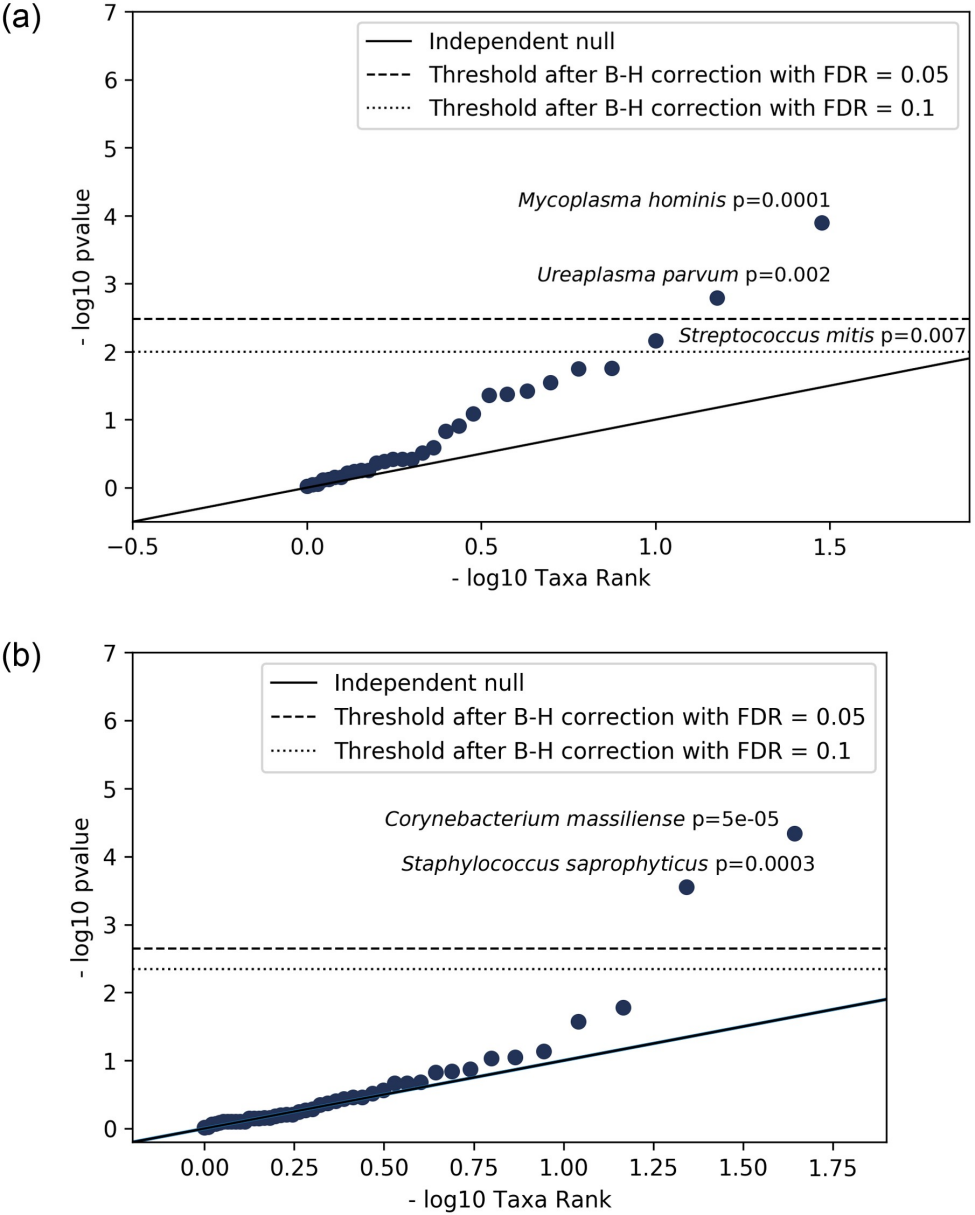
A. Three bacterial microorganisms significantly correlated between matched maternal and umbilical cord plasma. Correlations of rank of each microorganism across maternal plasma and matched umbilical cord plasma and their significances for individuals that had histological chorioamnionitis (n = 14). The solid line represents an independent null distribution, and the dashed and dotted lines represent the Benjamini-Hochberg correction with a false discovery rate of 0.05 and 0.1, respectively. B. Correlations of rank of each microorganism across maternal plasma and matched umbilical cord plasma and their significances for patients that had normal placental histology (n = 29). The solid line represents an independent null distribution, and the dashed and dotted lines represent the Benjamini-Hochberg correction with a false discovery rate of 0.05 and 0.1, respectively.

We next analyzed correlations between the maternal and umbilical cord plasma. We calculated the p-value of the Spearman correlation of the abundance of each microorganism across the maternal samples and its abundance in the paired umbilical cord plasma samples. Exact abundance may vary across samples due to background levels of environmental microbial DNA. Hence, the measure we used for this correlation was a p-value representing the significance of the enrichment of the microorganism above the expected background. We first confirmed that each microorganism was not affected by the lot change and, therefore, was not a false positive (see Methods). This method identified three microorganisms for removal: *Comamonas testosteroni*, *Ralstonia pickettii*, and *Cupriavidus metallidurans*. The abundance over all negative control samples is plotted in S1 Fig and a list of organisms identified is included in S1 and S2 Tables. We applied this analysis separately on the patients with positive and negative histologic chorioamnionitis, with the positive group expected to show evidence of infection and the negative group expected to represent controls. The p-values were compared to an independent null distribution and plotted in Fig 4A and 4B. Microorganisms that lie far above the line are the most significantly correlated and most likely to be causing chorioamnionitis.

In cases of histologic chorioamnionitis, the plot suggests a general correlation between maternal plasma and umbilical cord plasma and identifies three microorganisms, *Streptococcus mitis*, *Ureaplasma parvum*, and *Mycoplasma hominis* that show a strongly correlated signal in maternal and cord plasma. *M*. *hominis* and *U*. *parvum* are significant after Benjamini-Hochberg correction for multiple testing hypothesis with a false discovery rate (FDR) of 0.05, and *S*. *mitis* is significant only with an FDR of 0.1. This unbiased analysis suggests these organisms are likely causing chorioamnionitis in the cases in which they are present.

In the patients that had negative histology results, most microorganisms are only correlated as much as expected by chance and therefore match the null distribution. However, there were two microorganisms that showed stronger than expected correlations: *Corynebacterium massiliense* and *Staphylococcus saprophyticus*. *Staphylococcus saprophyticus* did not change abundance in the negative control samples, suggesting this could be a real signal. This pathogen is a known cause of urinary tract infections [34, 35], although its association with IAI or chorioamnionitis is less clear.

## Comment

### Principal findings of the study

We demonstrate that NGS can detect microbial cfDNA in the maternal plasma of women with clinical and/or histological chorioamnionitis. There were numerous differences noted between preterm and term pregnancies with certain organisms seen abundantly in preterm patients and not in at term healthy patients. Lactobacillus spp. were found to be more abundant only in at term pregnancies (Fig 2). Additionally, Ureaplasma urealyticum, Ureaplasma parvum, Mycoplasma hominis, and Haemophilus influenza were found almost exclusively in cases of chorioamnionitis (Figs 2 and 3). Streptococcus mitis, Ureaplasma parvum, and Mycoplasma hominis were significantly correlated between matched maternal and umbilical cord plasma samples, suggesting those organisms are causative pathogens (Fig 4A).

### Results in the context of what is known

This is the first study that utilized NGS to analyze maternal and umbilical cord plasma for microbial cfDNA. The majority of studies previously have relied on amniotic fluid cultures or molecular diagnostics of the amniotic fluid [36]. However, Oh et al. demonstrated that 24% of patient with clinical chorioamnionitis in preterm gestation have no evidence of intraamniotic inflammation or culture-proven IAI [17]. 66% (35/53) of patients within that study had negative amniotic fluid cultures which may demonstrate that standard culture techniques are not adequately identifying IAI [37]. Additionally, amniocentesis is an invasive procedure that does carry additional risks [38]. Our results identified microorganisms consistent with previous studies [13, 31–33], including organisms known to be specifically pathogenic in the setting of chorioamnionitis.

### Clinical implications

Plasma NGS of microbial cfDNA is a rapid and non-invasive test that is achievable with small quantities of plasma. Future utility of this method will depend on improvements in detecting clinically relevant infections in the maternal plasma samples. The ultimate goal of this method is to diagnose infections during pregnancy from maternal plasma. This would allow for targeted antibiotic therapy before and after delivery and improve upon current practices in which broad-spectrum antibiotics are utilized that disrupt the neonatal microbiome and have known toxic side effects. While this first study in cases of chorioamnionitis show a relevant biological signal of microbial cfDNA in maternal plasma, additional developments are required to evolve this RUO technology into a prenatal diagnostic test.

### Strengths and limitations

The strengths of this study include having a large matched set of maternal and umbilical cord plasma with well-documented clinical characteristics. Others strengths were the utilization of cfDNA rather than detection of whole organisms and the ability to use samples that were up to 8 years old.

The main limitation of this study is that samples were collected within larger observational studies that were not designed to measure performance of NGS, and specifically of this RUO test, in this population. Specifically, samples analyzed in this study did not have matched orthogonal confirmatory tests for IAI, such as culture and PCR of the amniotic fluid. Therefore, the main set of analyses we performed were aimed at finding concordance between maternal plasma and umbilical cord blood plasma and enrichment of previously known chorioamnionitis-causing microorganisms. This work identifies a biological signal of IAI but does not yet function as a diagnostic.

A major limitation with our study was the overall small number of cases of clinical or histological chorioamnionitis. Other limitations included the variability in sample collection inherent to observational studies, especially relative to antibiotic initiation, steroid therapy and latency until delivery. Additionally, abundance thresholds for clinical significance of each microorganism have yet to be established in this study population (although it has been established in the setting of sepsis, in which abundances are likely higher [39]). Microbial cfDNA in the plasma may be significantly increased or decreased based on the timing of antibiotic therapy, which antibiotic used and the timing of collection relative to delivery [40]. Furthermore, due to the nature and severity of complications in these cases, antibiotics are often initiated early and overall blood culture positivity rates are very low, which doesn’t allow for confirmation of the sequencing results.

## Conclusion

In conclusion, we report the first study demonstrating that NGS can detect microbial cfDNA in maternal plasma and umbilical cord plasma in patients with clinical or histological chorioamnionitis. These findings confirm the previously reported microorganisms in patients with clinical chorioamnionitis, IAI or neonatal sepsis. We also report a correlation between the abundance of microbial cfDNA in matched maternal and umbilical cord plasma. Prospective studies are needed to determine the utility of this test in the diagnosis and management of IAI.

## Supporting information

S1 Table Organisms identified in maternal blood plasma. (DOCX)

S2 Table Organisms identified in umbilical cord blood plasma. (DOCX)

S1 Appendix (DOCX)

S1 Fig Taxa for which the abundance in negative control samples changed significantly due to a reagent lot change were removed from the spearman correlation analysis and are shown here. To identify such taxa, we plotted and compared the mean abundance of each taxon before and after the lot change. If the mean abundance changed by a factor of at least 2 and a difference of at least 10 molecules per microliter, we removed the taxon from the analysis. This method identified microorganisms for removal that are plotted as shown.
(TIF)
