# Supplementary material for: Detection of microbial cell-free DNA in maternal and umbilical cord plasma in patients with chorioamnionitis using next generation sequencing
Source: PLoS One. 2020 Apr 15;15(4):e0231239. doi: 10.1371/journal.pone.0231239 (PMC7159194; doi:10.1371/journal.pone.0231239)
Supplement: S1 Table — (DOCX) [file pone.0231239.s001.docx]

**Supplementary Table 1. Organisms identified in maternal blood plasma**

| *Samples with histologic chorioamnionitis only* | *Samples with both histological and clinical chorioamnionitis* | *Samples with clinical chorioamnionitis only* |
| --- | --- | --- |
| *Escherichia coli* | ***Pseudomonas pseudoalcaligenes*** |  |
| *Helicobacter pylori* |  |  |
| *Mycoplasma hominis* |  |  |
