# Supplementary material for: Detection of microbial cell-free DNA in maternal and umbilical cord plasma in patients with chorioamnionitis using next generation sequencing
Source: PLoS One. 2020 Apr 15;15(4):e0231239. doi: 10.1371/journal.pone.0231239 (PMC7159194; doi:10.1371/journal.pone.0231239)
Supplement: S2 Table — (DOCX) [file pone.0231239.s002.docx]

**Supplementary Table 2. Organisms identified in umbilical cord blood plasma**

| *Samples with histologic chorioamnionitis only* | *Samples with both histological and clinical chorioamnionitis* | *Samples with clinical chorioamnionitis only* |
| --- | --- | --- |
| *Anaerococcus tetradius* | ***Citrobacter koseri*** | ***Lactobacillus crispatus*** |
| *Bacteroides fragilis* | ***Corynebacterium aurimucosum*** | ***Lactobacillus jensenii*** |
| *Camplyobacter hominis* | ***Corynebacterium urealyticum*** | ***Streptococcus anginosus*** |
| *Enterococcus faecalis* | ***Enterococcus faecalis*** |  |
| *Escherichia coli* | ***Finegoldia magna*** |  |
| *Fusobacterium nucleatum* | ***Klebsiella pneumoniae*** |  |
| *Kytococcus sedentarius* | ***Lactobacillus iners*** |  |
| *Methylobacterium mesophilicum* | ***Methylobacterium mesophilicum*** |  |
| *Micrococcus luteus* | ***Peptoniphilus harei*** |  |
| *Micrococcus lylae* | ***Prevotella bivia*** |  |
| *Mycoplasma hominis* | ***Prevotella intermedia*** |  |
| *Peptoniphilus harei* | ***Streptococcus mitis*** |  |
| *Prevotella buccalis* | ***Streptococcus pneumoniae*** |  |
| *Streptococcus pasteurianus* | ***Ureaplasma parvum*** |  |
| *Ureaplasma urealyticum* |  |  |
