# Supplementary material for: Detection of microbial cell-free DNA in maternal and umbilical cord plasma in patients with chorioamnionitis using next generation sequencing
Source: PLoS One. 2020 Apr 15;15(4):e0231239. doi: 10.1371/journal.pone.0231239 (PMC7159194; doi:10.1371/journal.pone.0231239)
Supplement: S1 Appendix — (DOCX) [file pone.0231239.s003.docx]

**Supplementary methods**

*Plasma next-generation sequencing (NGS) for pathogen detection*

Plasma NGS testing was performed at Karius, Inc (Redwood City, Calif), a reference laboratory with Clinical Laboratory Improvement Amendments certification and College of American Pathologists accreditation. This broad plasma NGS test was validated to detect >1000 microorganisms including bacteria, DNA viruses, and eukaryotic pathogens (yeasts, mold, and protozoa). For plasma NGS, whole blood (minimum of 4 mL) was collected in a plasma preparation tube by a peripheral blood draw. The sample was centrifuged at 1100 rcf for 10 minutes within 6 hours of collection to separate the plasma. The processed specimen was then shipped at ambient temperature to Karius, Inc.

*Sequencing*

The plasma sample was centrifuged at 16,000 rcf for 10 minutes at room temperature and spiked with a known concentration of synthetic DNA molecules for quality control purposes. Cell-free DNA was extracted from 0.5 mL of plasma by a magnetic bead-based method (Omega Bio-Tek, Norcross, Ga). DNA libraries for sequencing were constructed with a modified Ovation Ultralow System V2 library preparation kit (NuGEN, San Carlos, Calif). Negative controls (buffer only instead of plasma) and positive controls (healthy human plasma spiked with a known mixture of microbial DNA fragments) were processed alongside the sample. The sample was then multiplexed and sequenced on the NextSeq 500 (Illumina, San Diego, Calif).

*Bioinformatics pipeline analysis*

Primary sequencing output files were processed using bcl2fastq (v2.17.1.14) to generate the demultiplexed sequencing reads files. Reads were filtered on the basis of sequencing quality and trimmed on the basis of partial or full adapter sequence. The bowtie2 (version 2.2.4) method was used to align the remaining reads against human and synthetic molecule references. Sequencing reads exhibiting strong alignment against the human references or the synthetic molecule references were collected and filtered out from further analysis. Remaining reads were aligned against Karius' proprietary microorganism reference database using National Center for Biotechnology Information BLAST (version 2.2.30). A mixture model is used to assign a likelihood to the complete collection of sequencing reads that includes the read sequence probabilities and the (unknown) abundances of each taxon in the sample. An expectation-maximization algorithm is applied to compute the maximum likelihood estimate of each taxon abundance. The quantity for each organism identified is expressed in Molecules Per Microliter (MPM), the number of DNA reads from that particular organism per microliter of plasma.
