# Supplementary figures and images for: Detection of microbial cell-free DNA in maternal and umbilical cord plasma in patients with chorioamnionitis using next generation sequencing
Source: PLoS One. 2020 Apr 15;15(4):e0231239. doi: 10.1371/journal.pone.0231239 (PMC7159194; doi:10.1371/journal.pone.0231239)

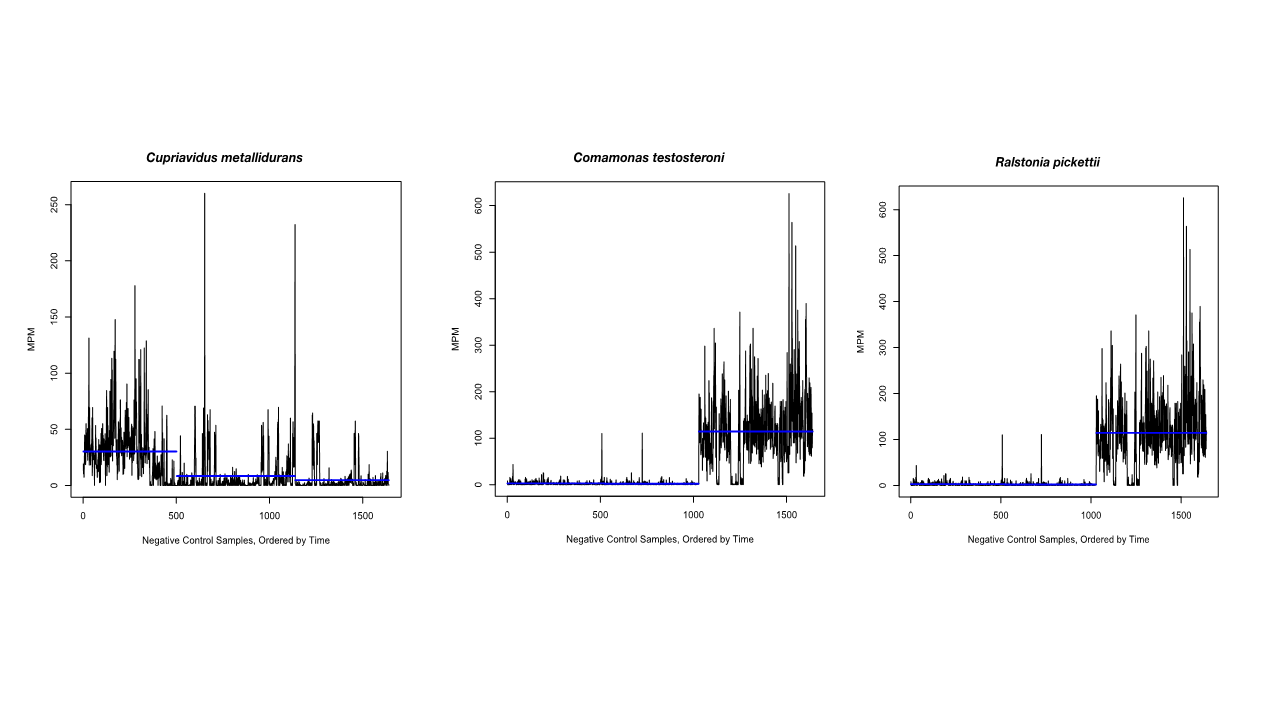

Supplement: S1 Fig — To identify such taxa, we plotted and compared the mean abundance of each taxon before and after the lot change. If the mean abundance changed by a factor of at least 2 and a difference of at least 10 molecules per microliter, we removed the taxon from the analysis. This method identified microorganisms for removal that are plotted as shown. (TIF) [file pone.0231239.s004.tif]
